# Perceived occupational stressors among emergency medical service providers: a qualitative study

**DOI:** 10.1186/s12873-021-00430-6

**Published:** 2021-03-23

**Authors:** Ali Afshari, Seyed Reza Borzou, Farshid Shamsaei, Eesa Mohammadi, Leili Tapak

**Affiliations:** 1 Nursing and Midwifery School, Hamadan University of Medical Sciences, Hamadan, Iran; 2 Chronic Diseases (Home Care) Research Center, Hamadan University of Medical Sciences, Hamadan, Iran; 3 Maternal and Child Care Research Center,Behavioral Disorders and Substance Abuse Research Center, Hamadan University of Medical Sciences, Hamadan, Iran; 4 Faculty of Medical Sciences, Nursing Department, Tarbiat Modares University, Tehran, Iran; 5 Department of Biostatistics, School of Public Health, Modeling of Noncommunicable diseases Research Center, Hamadan University of Medical Sciences, Hamadan, Iran

**Keywords:** Stress, Emergency medical services, Qualitative study

## Abstract

**Introduction:**

Emergency medical services (EMS) providers are at continuous exposure to occupational stressors which negatively affect their health and professional practice. This study explored perceived occupational stressors among EMS providers.

**Methods:**

This qualitative study was conducted from December 2019 to April 2020 using conventional content analysis. Sixteen EMS providers were purposively selected from EMS stations in Hamadan, Iran. Semi-structured interviews (with length of 45–60 min) were held for data collection. Data were analyzed through Graneheim and Lundman’s conventional content analysis approach.

**Findings:**

Data analysis resulted in the development of two themes, namely critical conditions of EMS provision, and personal and professional conflicts. The six categories of these two themes were complexity of patients’ clinical conditions, interruption of EMS provision, health hazards, interpersonal problems, interprofessional interactions, and legal conflicts.

**Conclusion:**

Besides the stress associated with emergency patient care, EMS providers face many different occupational stressors. EMS managers can use the findings of the present study to develop strategies for reducing occupational stress among EMS providers and thereby, improve their health and care quality.

## Introduction

Emergency medical services (EMS) are one of the main components of healthcare provision with significant contribution to the reduction of out-hospital mortality [1]. EMS providers provide their services to patients with critical conditions and in complex situations [2] which expose them to different unpredictable stressors and threats. Consequently, they experience high levels of stress and suffer from chronic stress. Studies showed that 22% of EMS providers deal with stress-related complications [3, 4] such as anxiety, irritability, social isolation, sleep disorder [5–8], job dissatisfaction, burnout [9–11], workplace incivility, leaving the profession, suicide [12–14] post-traumatic stress disorder, risk behaviors, psychological problems, and depression, and are more likely to commit medical errors [15–17].

Different factors can cause stress among EMS providers. These factors include placement in dangerous and uncontrollable conditions, distance from resources, witnessing heartbreaking senses of severe accidents [17], threats and violence, long shifts, short time for care provision [18], decision making under pressure, no control on the emergency scene [19], care provision to patients with critical conditions, witnessing patients’ death [20], the risk of infection transmission, and threats by mentally unstable patients [2].

Occupational stressors widely vary in different work conditions depending on the workplace characteristics, interpersonal relationships, culture, and social interactions [21]. Therefore, studies in different conditions and settings are needed to provide detailed information about occupational stressors. Although different quantitative studies have been conducted so far in Iran into EMS providers’ level of stress [22, 23], our literature search revealed no qualitative study into their perceived occupational stressors. The present study was conducted to address this gap. The aim of the study was to explore perceived occupational stressors among EMS providers.

## Methods

We selected a qualitative study design in which we used individual interviews with EMS providers in Hamadan, Iran. In reporting this qualitative study we have adhered to the COREQ (Consolidated criteria for reporting qualitative research) guidelines [24].

This qualitative study was conducted from December 2019 to April 2020 using conventional content analysis. As an analytical approach, content analysis provides a new insight for understanding the phenomenon of interest [25]. Participants were 16 EMS providers purposively selected from EMS stations in Hamadan, Iran. Emergency Medical Services in Iran was established in 1978. This organization is a subset of the Ministry of Health, Treatment and Medical Training. EMS delivery is financed by government and is free of charge. The EMS providers in Iran comprise a combination of nurses, and EMS technicians. The missions of the system are carried out by two EMS providers, and a resident physician in the dispatch center who provides telephone consultation to the EMS providers on the mission. Inclusion criteria were a work experience of more than 2 years in EMS system and agreement for participation. Despite considering the principles of purposeful sampling, we tried to select participants by using maximum variation sampling in terms of age, work experience, degree and place of work.

Data were collected through in-depth semi-structured face-to-face interviews held by the first author in a meeting room located at the central medical department. Initially, two pilot interviews were conducted to develop an interview guide. Three of the authors assessed the integrity and the accuracy of these two interviews and the interview guide was developed. These two interviews were not included in the final analysis. Then, main interviews were conducted using the guide. Examples of the interview questions were, “May you please talk about your occupational experiences?” “Can you explain about EMS missions with negative effects on you?” “Have you ever encountered tense situations at work?” “Can you explain your feelings in those situations?” Interviews lasted 45–60 min and were recorded using an MP3 player. Each interview was immediately typed word by word. Data collection was continued up to data saturation which was achieved after 12 interviews. Nonetheless, four more interviews were held to ensure data saturation and hence, data collection was finished after 16 interviews with 16 EMS providers. There were no participants who subsequently refused to participate, withdrew consent or dropped out. All interviews were conducted in Persian. Some of the participants’ phrases and speeches are semantically translated into English and given in this article. The English translation was reviewed and approved by a professional linguist.

Concurrently with data collection, the data were analyzed through Graneheim and Lundman’s five-step conventional content analysis approach [26]. The transcript of each interview was perused several times and primary codes were generated and categorized according to their similarities and differences. Then, categories with conceptual similarities were grouped into larger categories and main themes. The first author conducted the interviews. Three members of the research team coded the data. The collected data were analyzed in MAXQDA v. 2010.

Trustworthiness was established using Guba and Lincoln’s four criteria [27]. To ensure credibility, prolonged engagement with the data and member checking were used. After the extraction of the initial codes, the transcripts were made available to the participants to correct or confirm the meanings and initial codes. Confirmability was ensured through peer checking, in which two external peers experienced in qualitative research assessed some of the interviews and the corresponding codes and categories and confirmed the accuracy of data analysis. Dependability was also ensured through data analysis by three of the study authors who shared their results and reached agreement over data analysis in several meetings. Sampling with maximum variation, descriptions of participants’ characteristics, and comparison of the study findings with the findings of other studies were also performed to ensure transferability.

The Institutional Review Board and the Ethics Committee of Hamadan University of Medical Sciences, Hamadan, Iran, approved this study (code: IR.UMSHA.REC.1398.685). Before interviews, interviewees were verbally informed about the study aim and methods, confidential data management, and their freedom to voluntarily withdraw from the study. Written informed consent was obtained from each participant. This research was part of a PhD dissertation supported by the Hamadan University of Medical Sciences.

## Results

All participants were male because there was no female EMS provider in Hamadan’s emergency medical system. Fourteen participants were married and ten were working in urban EMS stations (Table 1). Their perceived occupational stressors were grouped into two main themes, namely critical conditions of EMS provision, and personal and professional conflicts (Table 2).

**Table 1:** Characteristics of the participants

Characteristics of participants	N (%) or range (Median)
Educational degree	
EMT	4 (25)
BS in EMS	3 (19)
BS in Nursing	4 (25)
MSc in Nursing	5 (31)
Years in Practice	4–28 (14)
Age	26–50 (36)
Gender	
Male	16 (100)
Marital Status	
Married	12 (75)
Single	4 (25)
Ambulance station location	
Urban	10 (62)
Rural	6 (38)

**Table 2:** Perceived occupational stressors among EMS providers

Themes	Categories	Subcategories
The critical conditions of EMS provision	Complexity of patients’ clinical conditions	- Encountering patients with emergency medical conditions
		- Limited professional knowledge and skills
	Interruption of EMS provision	- Unreasonable expectations of EMS providers
		- Troubles in EMS missions caused by lay people
	health hazards	- Threats at the emergency scene
		- Injuries related to violence
		- Ambulance crashes
Personal and professional conflicts	Interpersonal problems	- Incompatibility with colleagues
		- Ineffective familial role performance
	Interprofessional interactions	- Patient hospital admission
		- Interdisciplinary collaboration
	Legal conflicts	- Video recording of EMS activity
		- Legal prosecution against EMS providers

The following is a description of categories and subcategories with quotes. In this study verbatim quotations were used. Verbatim quotations are synonymous with direct quotations, which are a basic source of raw data in qualitative research that serve to reveal the informants’ emotions and experiences [28]. Also in reporting this qualitative study we have adhered to the COREQ guidelines [24].

### The critical conditions of EMS provision

Emergency medical services are provided in critical and complex unpredictable conditions and hence, EMS providers may face different problems in care provision. This main theme had three categories, namely complexity of patients’ clinical conditions, interruption of EMS provision, and health hazards.

#### Complexity of patients’ clinical conditions

In some missions, EMS providers may encounter patients with multiple injuries or critical conditions and hence, may need quick interventions in order to prevent further injuries or death. The two subcategories of this category were encountering patients with emergency medical conditions and limited professional knowledge and skills.

##### Encountering patients with emergency medical conditions

Participants noted that in some missions, they encounter patients with critical conditions who need multiple life support interventions. They noted that EMS provision to these patients puts them under high pressure.

The victim had several ruptures on the chest and abdomen due to knife attack. He had severe bleeding and hardly breathed. I had great stress because of the risk of cardiac arrest. (P9)

##### Limited professional knowledge and skills

Participants reported that Quality EMS provision requires adequate knowledge, skills, and experience regarding pre-hospital care. Therefore, inadequate knowledge, skills, and experience had caused stress for some participants.

The baby might be born at any moment. I hadn’t experienced such missions. I thought with myself what I could do if the baby was trapped in the birth canal. (P4)

#### Interruption of EMS provision

Respondents stated that although lay people in the emergency situations can help save patients’ lives, they may interrupt or postpone EMS provision. The two subcategories of this category were unreasonable expectations of EMS providers and troubles in EMS missions caused by lay people.

##### Unreasonable expectations of EMS providers

Participants noted that people at emergency scenes may expect unreasonable expectations of EMS providers and ask them to do things which are not in their job specifications.

The patient didn’t need transfer to hospital. However, his family members insisted on transfer. Such conditions cause me great stress. (P2)

##### Troubles in EMS missions caused by lay people

One of the main concerns of participants was related to delay in arriving at the emergency scene or transferring patients to hospital due to some drivers’ intentional blocking of ambulance.

We were going to the scene with ambulance siren. Suddenly, a car overtook us, moved in front of us, slowed down, and did risky movements which caused delay in our arrival at the scene. (P11)

#### Health hazards

Pre-hospital settings are unpredictable and uncontrollable and hence, health hazards are inevitable. The three subcategories of this category were Threats at the emergency scene, injuries related to violence, and ambulance crashes.

##### Threats at the emergency scene

Participants reported that some emergency scenes and situations are unpredictable and may include exposure to potential risks and threats which can cause injuries to EMS providers.

A car rollover victim had been trapped in the car. While we were taking him out, my arm hit to the rough edge of the car door, resulting in a relatively deep rupture. (P8)

##### Injuries related to violence

Participants noted that one of their main occupational stressors is inappropriate and violent behaviors of people who attend emergency scenes.

While managing the bleeding, the patient experienced cardiac arrest. During resuscitation, one of his friends angrily accused us of his death, justifying that he was alive until several minutes ago. He finally punched me in the face. (P6)

##### Ambulance crashes

Participants mentioned that driving ambulance in emergency situations exposes them to significant injuries and puts them at risk for car accidents.

While we were returning from a mission in a snowy weather, our ambulance swerved off the road due to road slipperiness and turned over. I experienced several wounds in my face and my colleague experienced elbow fracture. (P9)

### Personal and professional conflicts

Personal and professional conflicts were the second main occupational stressor for EMS providers. The three categories of this theme were interpersonal problems, interprofessional interactions, and legal problems.

#### Interpersonal problems

Interpersonal problems with family members and colleagues can cause EMS providers varying levels of stress. The two subcategories of this category were incompatibility among colleagues and ineffective familial role performance.

##### Incompatibility among colleagues

Participants noted that incompatibilities among EMS providers regarding their personality characteristics, skills, and experiences can cause them occupational stress.

I was dispatched on a mission with a newly employed colleague. The patient needed resuscitation and advanced life support interventions. I had high level of stress because I had to do most care procedures myself. (P12)

##### Ineffective familial role performance

Participants reported that because of long shifts and working in remote EMS stations located in roads, EMS providers were far from their family members and were unable to effectively perform their familial roles.

Most household and childrearing responsibilities are on my wife’s shoulders due to my work conditions. It seems that my wife hasn’t so far coped with my work and cannot understand me. (P5)

#### Interprofessional interactions

Pre-hospital EMS provision requires close interprofessional interactions. Consequently, ineffective interprofessional interactions can be a source of stress for EMS providers. The two subcategories of this category were patient hospital admission and interdisciplinary collaboration.

##### Patient hospital admission

Participants highlighted that they sometimes experience problems while delivering patients to hospitals, particularly in case of patients with critical conditions.

We transferred a patient with chest pain to hospital. The emergency room nurse said that only patients with cardiac problems were admitted and asked us to wait until they took an electrocardiogram strip and laboratory tests in order to ensure that the patient’s problem was related to heart and then, decide on admission. (P4)

##### Interdisciplinary collaboration

Participants reported that some missions require the involvement and collaboration of different staff such as firefighters, EMS providers, and police officers. However, in some cases, ineffective interdisciplinary collaboration causes EMS staff some levels of stress.

The victim had been trapped in the care and firefighting equipment was needed for releasing the patient. I contacted the firefighting station and informed them about the situation. We were under pressure because people in the scene considered us responsible for saving the patient. (P10)

#### Legal conflicts

Working in the frontline of care provision, complex conditions, and public areas can cause some legal problems for EMS providers. The two subcategories of this category were Video recording of EMS activity and legal prosecution against EMS providers.

##### Video recording of EMS activity

Some participants stated that while providing rescue, some people at the scene of the accident, both tangible and intangible, recording these activities. This action causes stress to EMS providers by invading the privacy of EMS providers and patients, and disrupting the focus on medical procedures.

A patient had experienced cardiac arrest in a bus station. After the first cycle of chest compression, I noticed that some people were filming us with mobile phones. (P8)

##### Legal prosecution against EMS providers

Respondents stated that some EMS providers may be legally prosecuted for their practice. Participants reported that such prosecutions and the necessity to attend juridical settings are highly stressful for them.

In a mission, we transferred a girl who had committed suicide by taking many unknown pills. We transferred her to hospital. She died two weeks later. Her family made a legal claim against us. (P3)

## Discussion

This study aimed to explore perceived occupational stressors among EMS providers. Findings revealed that the main perceived occupational stressors among EMS providers were critical conditions of EMS provision, and personal and professional conflicts. These findings are discussed in what follows.

### Critical conditions of EMS provision

Study findings showed that patient’s critical conditions, their death during EMS missions, and EMS providers’ lack of knowledge and skills regarding some aspects of EMS provision are among the main occupational stressors for EMS providers. In line with these findings, former studies reported that the main occupational stressors for EMS providers were care provision to patients with critical conditions [29] and patients’ death during EMS provision [4]. Patients with critical conditions are at high risk for death during transfer to hospital settings [30]. Our findings also showed that EMS providers experienced stress and anxiety in new EMS missions which they had limited experience about. Adequate knowledge and experience are among the main requirements for sound decision making in emergency situations because EMS providers have not the permission to use the trial and error method during their missions [31]. In line with our findings, a former study showed that due to their limited decision making skills, EMS providers with limited professional experience undergo higher levels of stress [32]. Our participants also referred to any factor which delayed and interrupted their care provision as an occupational stressor. Lay people’s interventions before ambulance arrival and during EMS provision can also negatively affect care outcomes and cause stress for EMS [29]. It seems that the reasons for lay people’s interference with EMS provision are their lack of knowledge about EMS and their excessive empathy with patients and victims. Health hazards were the third main perceived occupational stressor among EMS providers in the present study. Findings showed that most participants had experienced physical injuries due to the environmental conditions of the emergency scenes, people’s violent behaviors, and ambulance accidents. In most missions, EMS providers work in uncontrolled situations without knowing potential threats [33]. Some of these threats include the risk of contact with sharp things, exposure to chemicals, and electric shock [34]. Some threats are inseparable parts of EMS missions, while some of them can be prevented by the cooperation and coordination of relief and security teams (Firefighters, Red Crescent and Police). In recent years, the rate of violence against EMS providers has increased [35, 36] so that a study reported that 66% of them had experienced instances of violent behaviors and violence-related injuries [37]. EMS providers are in the frontline of care provision to patients, where patients’ family members may not be in good and stable mental conditions. Therefore, they are at great risk for threat and violence [13]. Another potential source of physical injury to EMS providers is related to ambulance crashes [38–40]. Compared with non-emergency situations, driving an ambulance in emergency situations increases the risk of fatal accidents by two times, the risk of serious injuries by eight times, and the risk of vehicle and equipment damages by 17 times even when using ambulance siren [41, 42]. Our findings showed that these factors are a major source of stress for EMS providers, while previous studies did not report any findings in this area. It seems that great focus on care provision and timely arrival at emergency scene or hospital as well as the negative effects of heartbreaking scenes disturbs EMS providers’ concentration on driving.

### Personal and professional conflicts

Study findings showed that EMS staff experienced different levels of stress due to personal and professional conflicts such as long shifts and work-family problems. In line with this finding, a former study showed that EMS providers and their family members suffered from stress due to work-family conflicts [43]. Another source of occupational stress in the present study was interpersonal conflicts among EMS colleagues and ineffective interpersonal interactions with rescue, relief, and hospital staff. Pre-hospital EMS provision requires close collaboration among EMS providers [44]. A study showed ineffective interdisciplinary collaboration as a main barrier to EMS provision in pre-hospital settings and recommended the use of a joint dispatch system for closer interdisciplinary collaboration [45]. We also found legal conflicts, such as legal prosecution, as a major source of severe stress for EMS providers. A study reported that most important sources of legal prosecution against EMS providers were patients (53%), healthcare providers (19%), and patients’ companions (12%) and the most important reasons of such prosecution were EMS providers’ limited professional skills (20%), transfer-related problems (18%), and loss of patients’ personal items (13%) [46]. EMS providers face irrational expectations of patients’ companions and hence, are at risk for legal prosecutions. We also found that EMS providers experienced stress because of video recording by public during relief and rescue. This problem was a stressful issue among some participants in the present study. Although video recording can be a useful tool for some purposes, it also has concerns in clinical settings. One concern is that videos invade the privacy of patients and health care professionals. In addition, professionals may fear that video data might be used for controlling purposes [47, 48].

## Conclusion

This study shows that a wide range of personal, interpersonal, organizational, and social factors can cause occupational stress for EMS providers. More quantitative and qualitative research is needed to better understand the stressors in the prehospital emergency field and its impact on EMS providers’ health and performance. EMS managers can use the findings of the present study to develop strategies for managing occupational stressors among EMS providers and thereby, improve the quality of EMS provision. These strategies may include periodical assessment of EMS providers for stress, offering stress management courses for them, provision of psychological and legal counseling to them, arrangement of interprofessional meetings, and establishment of a joint dispatch center and system. It is recommended that police station, fire department, and EMS organizations be involved in establishing a joint system and a joint rescue center. This system improves relief and rescue by correctly identifying and prioritizing the dispatch of specialized personnel related to the scene of accident.

### Limitations

This study has some limitations. While qualitative studies requires smaller sample sizes to allow for the collection of data about perception and the complex contextual factors that shape it, the generalizability may be limited. The present study was conducted in one of the cities in western Iran, and data were obtained from 16 EMS providers. Thus, more extensive studies with greater number of participants are required for generalizability of the results. Another limitation was the absence of female EMS providers in Hamadan’s emergency medical system, which could have affected the results, given their different perceptions of stressors. We were also unsure if participants were involved with another health care profession and results may have been influenced by previous experiences.

## Data Availability

The datasets analyzed during the current study are available from the corresponding author on request.

## Abbreviation

EMS: Emergency medical services
